# RNA-Sequencing based analysis of bovine endometrium during the maternal recognition of pregnancy

**DOI:** 10.1186/s12864-022-08720-4

**Published:** 2022-07-07

**Authors:** Bindu Adhikari, Chin N. Lee, Vedbar S. Khadka, Youping Deng, Glen Fukumoto, Mark Thorne, Kyle Caires, Jenee Odani, Birendra Mishra

**Affiliations:** 1 Department of Human Nutrition, Food and Animal Sciences, University of Hawaii at Manoa, Honolulu, HI 96822 USA; 2 Department of Quantitative Health Sciences, University of Hawaii at Manoa, Honolulu, HI 96822 USA

**Keywords:** RNA-Sequencing, Beef cattle, Endometrium, Maternal recognition, Early pregnancy

## Abstract

**Background:**

Maternal recognition is the crucial step for establishing pregnancy in cattle. This study aims to identify endometrial genes and biological pathways involved in the maternal recognition of pregnancy. Caruncular endometrial tissues were collected from Day 15–17 of gestation (pregnant), non-pregnant (absence of conceptus), and cyclic (non-bred) heifers.

**Results:**

Total RNAs were isolated from the caruncular endometrial tissues of pregnant, non-pregnant, and cyclic heifers, and were subjected to high-throughput RNA-sequencing. The genes with at least two-fold change and Benjamini and Hochberg *p*-value ≤ 0.05 were considered differentially expressed genes and further confirmed with quantitative real-time PCR. A total of 107 genes (pregnant vs cyclic) and 98 genes (pregnant vs non-pregnant) were differentially expressed in the pregnant endometrium. The most highly up-regulated genes in the pregnant endometrium were *MRS2*, *CST6*, *FOS*, *VLDLR*, *ISG15, IFI6, MX2*, *C15H11ORF34*, *EIF3M*, *PRSS22*, *MS4A8*, and *TINAGL1*. Interferon signaling, immune response, nutrient transporter, synthesis, and secretion of proteins are crucial pathways during the maternal recognition of pregnancy.

**Conclusions:**

The study demonstrated that the presence of conceptus at Day 15–17 of gestation affects the endometrial gene expression related to endometrial remodeling, immune response, nutrients and ion transporters, and relevant signaling pathways in the caruncular region of bovine endometrium during the maternal recognition of pregnancy.

**Supplementary Information:**

The online version contains supplementary material available at 10.1186/s12864-022-08720-4.

## Introduction

Beef cattle production is an important source of protein to meet the nutritional needs of a growing population. Improvements to beef cattle reproduction can help increase beef production worldwide to meet the increasing demand [1–3]. Early pregnancy failure is one of the critical factors that affect the economic output of the beef industry [4]. In ruminants, the successful establishment of pregnancy requires an intricate dialogue between the uterus and growing conceptus. The majority of pregnancy losses occur in the first month, especially around Day-19 of gestation, mainly due to the inability of the uterus to support conceptus growth and development or poor embryonic development. Understanding uterine changes during early pregnancy provide critical insight into reproductive success.

The endometrium undergoes dynamic changes during the peri-implantation period and provides the biological environment for embryonic growth and development [5]. The bovine endometrium consists of caruncles (aglandular) and intercaruncular tissue (glandular). The caruncle develops the vascular bed and is the site for embryo implantation and metabolic exchange. The endometrial secretions that support conceptus elongation are produced from the luminal and glandular epithelium [6]. In the ruminant, the fertilized oocyte undergoes a series of morphological and biochemical changes as a conceptus in the oviduct and uterus and begins to elongate between Days 12–14 of gestation [7]. By Day 15–17 of gestation, the conceptus develops into a filamentous form and produces interferon tau (IFNτ), which acts as the signal for the maternal recognition of pregnancy [8, 9]. The conceptus derived IFNτ promotes the persistence of the corpus luteum required for adequate progesterone production [10]. It is well-known that progesterone induces endometrial transcriptomes during the peri-implantation periods [10–12]. IFNτ also induces endometrial genes and proteins required for immunomodulation, extracellular matrix remodeling, and implantation-specific molecules [13, 14]. Recent studies have suggested that bovine embryos around the peri-implantation period induce endometrial gene expression in the intercaruncular region required to establish gestation [4, 7, 15]. Despite these studies, the transcriptomic changes in the caruncular portion of endometrial tissue during the maternal recognition of pregnancy (Day 15–17 of gestation) are not completely understood. Most previous studies have investigated the conceptus-induced gene expression in the caruncular and intercaruncular endometrial tissues and compared it with cyclic cows around the peri-implantation period [16–18], and yet, caruncular endometrial transcriptomes involved in the maternal recognition of pregnancy are not clearly understood. To further improve the conception rate in cattle, the knowledge of specific genes, proteins, and biological pathways during the maternal recognition of pregnancy is required throughout the uterus. As Day-15–17 of gestation is a critical period for the maternal recognition and establishment of pregnancy, we hypothesized that RNA-Sequencing based analysis of bovine caruncular endometrial tissues during the maternal recognition of pregnancy (Day-15–17 of gestation) would reveal important genes and biological pathways required for the maternal recognition of pregnancy. This study analyzed the genes and biological pathways in the caruncular endometrium during the maternal recognition of pregnancy (pregnant vs. cyclic) and (pregnant vs. non-pregnant) using RNA-Sequencing, and Real-time PCR (qPCR).

## Methods

### Animals and sampling

Animal husbandry, management, and handling procedures were under the Guide for the Care and Use of Agricultural Animals in Research and Teaching (Ag Guide 2020) [19]. Angus heifers (2–3 years old; *n* = 21) grazing Kikuyu grass *(Pennisetum clandestinum*) and Pangola (*Digitaria eriantha*) pastures were used for sampling. The estrous cycles of the heifers (*n* = 21) were synchronized using 25 mg of prostaglandin F2 alpha (PGF2 α; Lutalyse®, Zoetis, Parsippany, New Jersey, USA) administered intramuscularly on Day-1 (Day-1 designated as the first dose of PGF2 α) and Day-11 (Day-11 designated as the second dose of PGF2 α). Day Fifteen heifers were bred after detecting estrus. Cows were identified as pregnant (presence of conceptus) or non-pregnant (absence of conceptus). After incision of the uterus, the lumen of the uterus was exposed. Caruncles were identified as the small protuberances from the surface of endometrium, and carefully collected the protruded endometrial areas (4–5/heifer) as previously collected [20]. Caruncular endometrial tissues were collected on Day 15–17 of gestation (pregnant; *n* = 8) or absence of conceptus (non-pregnant; *n* = 7) or non-bred heifers (cyclic; *n* = 6) and were stored at -80 °C until further use.

### RNA isolation and quality control

Total RNA was isolated from frozen tissues (60–100 mg) using TRIzol reagent (Invitrogen, Carlsbad, CA) according to the manufacturer’s instructions as previously described [21]. The total RNA concentration was determined using NanoDrop One (Thermo Fisher Scientific, Madison, WI). RNA quality was determined with the Agilent 2100 Bioanalyzer (Agilent Technologies, Massy, France). The samples with an RNA integrity number (RIN) > 7 were further used for RNA-Sequencing and quantitative real-time PCR. The RNA was stored at -80 °C until further use for RNA-sequencing and quantitative real-time PCR.

### Library preparation and RNA sequence analysis

RNA-Seq libraries were prepared and sequenced at the University of Hawaii Cancer Center Genomics and Bioinformatics Shared Resource (UHCC GBSR) facility. A TruSeq Stranded mRNA kit (Illumina, San Diego, CA) was used to prepare the RNA-Seq libraries from total RNA samples extracted from bovine endometrium, including pregnant (*n* = 5), non-pregnant (*n* = 5), and cyclic (*n* = 5). Libraries were prepared according to the manufacturer’s protocol as previously described [21].

Data analysis of the RNA sequences were done at the University of Hawaii John A. Burns School of Medicine Bioinformatics Core Facility. Single-end reads in the FASTQ format were explored using FastQC (Babraham Institute, Cambridge, UK) and cleaned using Prinseq, a Perl script [22, 23]. The cleaning procedure included trimming low-quality reads from both 3’ and 5’ ends until a base pair of Phred quality score of 30 (99.9% accurate) or greater was found and filtering out reads having a mean quality score less than 30 and length below 30 nucleotides. Cleaned reads were aligned against the bovine reference genome (Bos_taurus.ARS-UCD1.2) using HiSAT2. The resulting SAM files were sorted, and converted to BAM files using SAMtools. Read counts mapped to bovine gene models were generated using htseq-count script from HTSeq package. Finally, bioconductor DESeq2 was used to get the differentially expressed genes among pregnant vs. non-pregnant (P vs. NP), pregnant vs. cyclic (P vs. C), and non-pregnant vs. cyclic (NP vs. C) groups (*n* = 4/group). In RNA Sequencing, genes having fold change (FC) greater than 2 in the endometrial sample and Benjamini and Hochberg q-value < 0.05 were considered differentially expressed.

### Pathways analyses

The ingenuity pathway analysis (IPA) is a human genome-based powerful search tool with several advanced functions that allows insightful data analysis and interpretation. The differentially expressed genes (DEGs) were subjected to the IPA (QIAGEN, Inc., https://www.qiagenbioinformatics.com/products/ingenuity-pathway-analysis) to gain insights into the canonical pathways and network discovery.

#### Functional annotation and gene ontology enrichment analysis

Functional and pathway analysis was carried out using an open web source named Enrichr (https://maayanlab.cloud/Enrichr/) to gain insight into the various Gene Ontology (GO) terms of the genes in bovine endometrium. The official gene symbol of the up-regulated genes was uploaded to the functional annotation tool in the Enrichr system, and the Bos taurus was selected as the reference genome. The genes that matched up with the genes in Enrichr were annotated into three GO terms: biological process, cellular component, and molecular function. All the GO terms were considered enriched at a modified *P*-value < 0.05 and a threshold gene count of 2.

#### Kyoto Encyclopedia of Genes and Genomes (KEGG)

The pathways enrichment for the up-regulated genes in the bovine endometrium using the Kyoto Encyclopedia of Genes and Genomes were analyzed [24]. The official gene symbol of the up-regulated genes was uploaded to the functional annotation tool in the Enrichr system, and the bovine was selected as the reference genome. The enrichment parameters were set to a threshold gene count of 2 and a modified Fisher Exact *P*-value < 0.05. The over-represented KEGG pathways terms were considered as enriched KEGG pathways.

### Quantitative real-time PCR (qPCR)

Among the most highly up-regulated genes, fourteen candidate genes (*MRS2, CST6, FOS, VLDLR, ISG15, IF16, MX2, C15H11ORF34, EIF3M, PENK, PRSS22, MS4A8, TINAGL1*, and *R3HDM1*) were selected for validation using qPCR. Primers specific to each gene were designed using the NCBI primer blast tool (Supplementary Table S1). Total RNA (1 μg) was reversed transcribed into cDNA using the High-Capacity cDNA Reverse Transcription Kit (Applied Biosystems, Foster City, California, USA). The newly synthesized cDNA (20 μL) was diluted (20X), and 3 μl per qPCR reaction was used.

The qPCR assay was performed in a 10 μL reaction mixture containing 3 μL of cDNA and 7 μL of PCR mix using QuantStudio™ 3 System (Applied Biosystems). The PCR mixture was prepared by adding 5 μL of PowerUp SYBR Green Master Mix (Applied Biosystems) and 1 μL each of forward and reverse primers specific to the target gene. The PCR mix and cDNA samples were loaded into a 96-well optical plate and were sealed with clear optical adhesive films (Applied Biosystems) as previously described [21]. The specificity of each primer was validated by running the melting curve, and qPCR products were assessed using gel electrophoresis. To determine the most stable housekeeping gene in the endometrial tissues, the expression of glyceraldehyde 3-phosphate dehydrogenase (GAPDH), beta-actin (β-actin), and TATA-Box Binding Protein (TBP) were analyzed in triplicates across the samples. β-actin was the most uniform housekeeping gene. The target genes were analyzed in triplicates, and the expression level was determined using the cycle threshold (Ct) values after normalization with β-actin. The fold change for each gene was calculated using the comparative CT method (2^−ΔΔCt^ method) [25]. Data for fold change were presented as a mean ± standard error on the bar diagram. Values were subjected to a one-way analysis of variance (ANOVA) followed by the Tukey HSD test for mean separation and comparison to determine differences between the treatments using R Studio. Differences were considered significant at a *p*-value < 0.05.

## Results

### RNA sequencing-based differentially expressed genes in the pregnant bovine endometrium during the maternal recognition of pregnancy

**Fig. 1:**
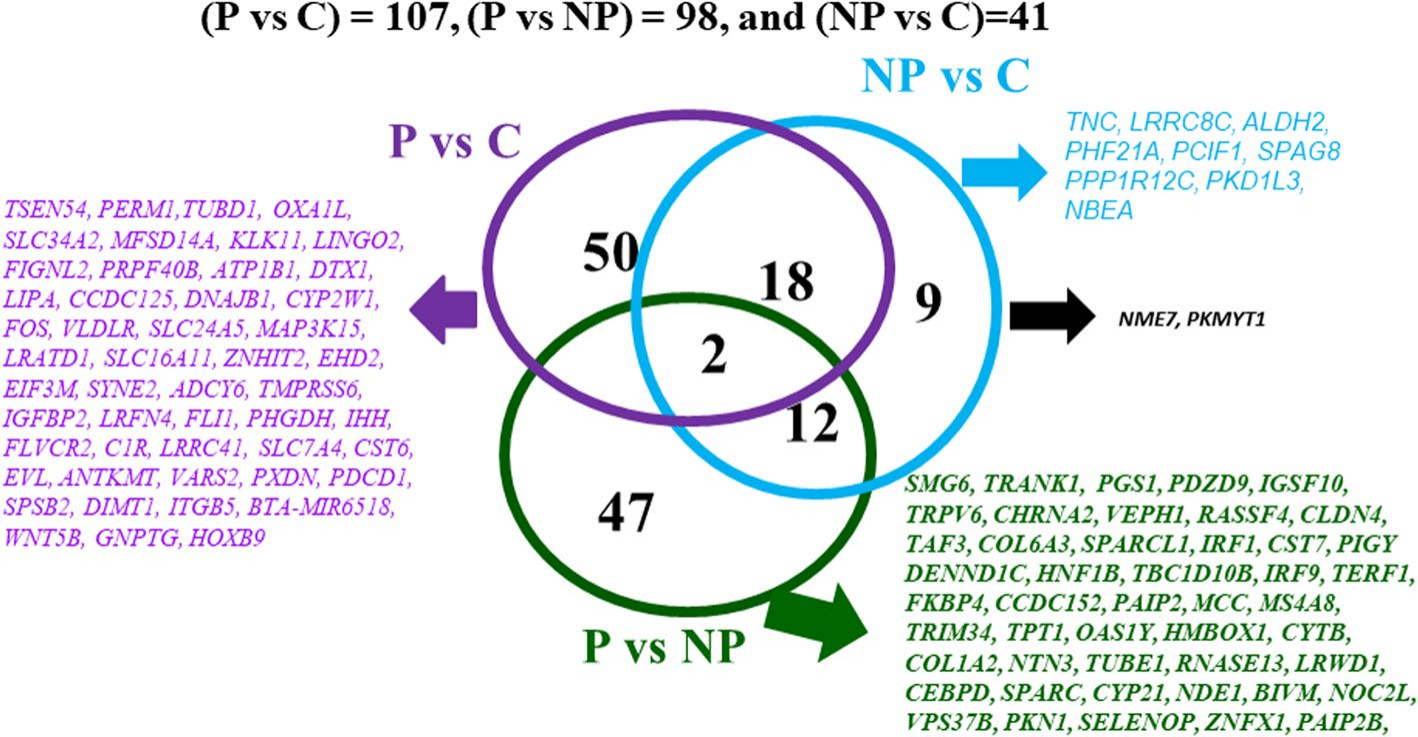
Venn diagram comparing the number of identified genes in the endometrium of pregnant (P) vs. cyclic (C), pregnant (P) vs. non-pregnant (NP), and non-pregnant (NP) vs. cyclic (C) cows along with the list of the name of the genes. Number of differentially expressed genes (DEGs) in P vs. C (*n* = 107), P vs. NP (*n* = 98), and NP vs. C (*n* = 41)

**Fig. 2:**
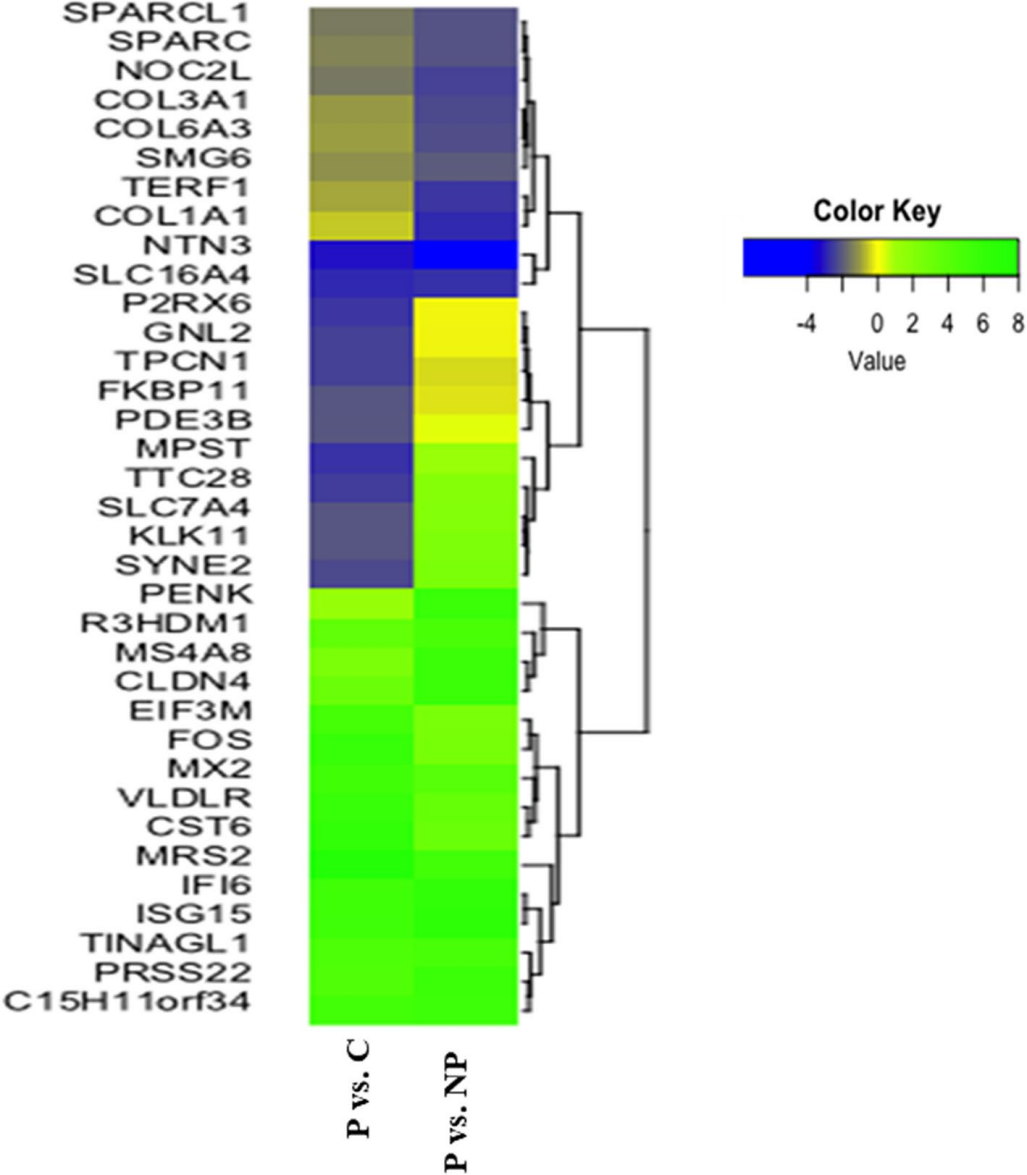
Heat-map of the 35 up-regulated genes in the bovine endometrium compared with pregnant versus non-pregnant (P vs. NP) and pregnant versus cyclic (P vs. C). Extremes of green color indicate the gene's higher expression, while blue color indicates a lesser expression of genes

Using RNA-Seq, the transcriptomics profile was analyzed in pregnant endometrium (Day 15–17 of gestation) compared to non-pregnant and cyclic cows. Raw sequencing reads in the FASTQ format were obtained from the replicated RNA-Seq libraries and evaluated for their qualities using FastQC. There was an average of 19.5 M, 23.6 M, and 20.6 M original raw reads in pregnant (P), non-pregnant (NP), and cyclic (C) cows, respectively. All the groups (P, NP, and C) had excellent quality sequences (> 96%). Mapping results of the bovine genome database showed that an average of 93.3% of the retained reads from pregnant, 94.3% from non-pregnant, and 94.2% from cyclic were uniquely mapped. A total of 27,270 gene transcripts were annotated using the Ensemble alignment of the bovine genome assembly. Differential expression analysis between pregnant, non-pregnant, and cyclic cows was conducted (DESeq2). A total of 98 genes were differentially expressed between pregnant and non-pregnant cows, 107 genes were differentially expressed (DE) between pregnant and cyclic, and 41 genes were differentially expressed between cyclic and non-pregnant (Fig. 1). Of the 98 genes found in non-pregnant endometrium, 47.9% (47) were uniquely expressed in pregnant cattle, and of the 107 DE genes in pregnant cattle, 46% (50) were uniquely expressed in pregnant cattle (Fig. 1). Only 23% of DE genes were shared between pregnant and non-pregnant cattle (Fig. 2).

To characterize the conceptus-induced transcriptomic changes in the pregnant endometrium, we shorted out the most highly up-regulated or down-regulated genes in the pregnant endometrium compared to cyclic and non-pregnant. The highly up-regulated genes in the pregnant endometrium (vs. cyclic) are *MRS2, CST6, FOS, VLDLR, ISG15, IF16, MX1, MX2, C15H11ORF34, EIF3M,* and *TINAGL1* (Table 1). Among these genes, *MRS2* was most highly expressed followed by *CST6, FOS, VLDLR*, and *ISG15*. The highly down-regulated genes in the pregnant endometrium (vs. cyclic) are *LRFN4, PKMYT1, ZNHIT2, VARS2, ARL2BP, LINGO2, SLC16A11, SLC16A4, TMEM151B*, and *NME7* (Table 2). Among these genes, *LRFN4* was highly down-regulated followed by *PKMYT1, ZNHIT2, VARS2*, and *ARL2BP*. The highly up-regulated genes in the pregnant endometrium (vs. non-pregnant) are *ISG15, IFI6, PENK, PRSS22, MS4A8, CLDN4, C15H11ORF34, MRS2, TINAGL1*, and *R3HDM1* (Table 3). Among these genes, ISG*15* was highly expressed followed by genes *IFI6, PENK, PRSS22,* and *MS4A8*. The highly down-regulated genes in the pregnant endometrium (vs. non-pregnant) are *SNX20, PACSIN1, NTN3, COL1A1, SLC16A4, TERF1, NOC2L, COL3A1, COL6A3,* and *SPARCL1* (Table 4). Among these genes, *SNX20* was highly down-regulated followed by *PACSIN1, NTN3, COL1A1*, and *SLC16A4*. However, some of the genes such as *ISG15, IFI6, MRS2, MX2, C15H11ORF34, and TINAGL1* were common in the pregnant endometrium as compared to both cyclic and pregnant endometrium indicating the crucial involvement of these genes in the maternal recognition of pregnancy*.*

**Table 1:** Up-regulated genes in the bovine pregnant endometrium (P vs. C)

S.N	Gene	Gene Description	Fold Change (log2)	padj
1	*MRS2*	Magnesium Transporter *MRS2*	7.727	0.000
2	*CST6*	Cystatin E/M	6.306	0.001
3	*FOS*	FOS Proto-Oncogene, AP-1 Transcription Factor Subunit	5.760	0.045
4	*VLDLR*	Very Low-Density Lipoprotein Receptor	5.691	0.008
5	*ISG15*	*ISG15* Ubiquitin Like Modifier	5.314	0.012
6	*IFI6*	Interferon Alpha Inducible Protein 6	5.215	0.000
7	*MX2*	MX Dynamin Like GTPase 2	5.189	0.018
8	*C15H11ORF34*	Placenta Expressed Transcript 1	5.107	0.006
9	*EIF3M*	Eukaryotic Translation Initiation Factor 3 Subunit M	4.973	0.003
10	*TINAGL1*	Tubulointerstitial Nephritis Antigen Like 1	4.489	0.000
11	*PXDN*	Peroxidasin	4.489	0.045
12	*PRSS22*	Serine Protease 22	4.442	0.039
13	*TUBD1*	Tubulin Delta 1	4.376	0.049
14	*TMPRSS2*	Transmembrane Serine Protease 2	4.348	0.000
15	*C1R*	Complement C1r	3.698	0.006
16	*FLVCR2*	Feline Leukemia Virus Subgroup C Cellular Receptor Family	3.692	0.013
17	*SLC2A1*	Solute Carrier Family 2 Member 1	3.688	0.002
18	*R3HDM1*	R3H Domain Containing 1	3.490	0.045
19	*MX1*	MX Dynamin Like GTPase 1	3.481	0.000
20	*BCAM*	Basal Cell Adhesion Molecule (Lutheran Blood Group)	3.468	0.001

**Table 2:** Down-regulated genes in the bovine pregnant endometrium (P vs. C)

S.N	Gene	Gene description	Fold Change (log2)	padj
1	*LRFN4*	Leucine-Rich Repeat and Fibronectin Type III Domain Containing	-3.276	0.000
2	*PKMYT1*	Protein Kinase, Membrane Associated Tyrosine/Threonine 1	-3.202	0.000
3	*ZNHIT2*	Zinc Finger HIT-Type Containing 2	-3.182	0.000
4	*VARS2*	Valyl-TRNA Synthetase 2, Mitochondrial	-3.167	0.001
5	*ARL2BP*	ADP Ribosylation Factor Like GTPase 2 Binding Protein	-3.141	0.000
6	*LINGO2*	Leucine-Rich Repeat and Ig Domain Containing 2	-3.104	0.000
7	*SLC16A11*	Solute Carrier Family 16 Member 11	-3.005	0.001
8	*SLC16A4*	Solute Carrier Family 16 Member 4	-2.861	0.001
9	*TMEM151B*	Transmembrane Protein 151B	-2.795	0.000
10	*NME7*	NME/NM23 Family Member 7	-2.743	0.000
11	*MPST*	Mercaptopyruvate Sulfur transferase	-2.672	0.000
12	*P2RX6*	Purinergic Receptor P2X 6	-2.636	0.000
13	*TTC28*	Tetratricopeptide Repeat Domain 28	-2.490	0.000
14	*GNL2*	G Protein Nucleolar 2	-2.454	0.000
15	*TPCN1*	Two Pore Segment Channel 1	-2.444	0.000
16	*SYNE2*	Spectrin Repeat Containing Nuclear Envelope Protein 2	-2.292	0.000
17	*FKBP11*	FKBP Prolyl Isomerase 11	-2.106	0.000
18	*SLC7A4*	Solute Carrier Family 7 Member 4	-2.096	0.000
19	*KLK11*	Kallikrein Related Peptidase 11	-2.095	0.001
20	*PDE3B*	Phosphodiesterase 3B	-2.076	0.000

**Table 3:** Up-regulated genes in the bovine pregnant endometrium (P vs. NP)

S.N	Gene	Gene Description	Fold Change (log2)	padj
1	*ISG15*	*ISG15* Ubiquitin Like Modifier	6.520	0.001
2	*IFI6*	Interferon Alpha Inducible Protein 6	6.300	0.000
3	*PENK*	Proenkephalin	5.578	0.000
4	*PRSS22*	Serine Protease 22	5.565	0.010
5	*MS4A8*	Membrane Spanning 4-Domains A8	5.560	0.012
6	*CLDN4*	Claudin 4	5.526	0.006
7	*C15H11ORF34*	Placenta Expressed Transcript 1	5.379	0.003
8	*MRS2*	Magnesium Transporter MRS2	5.096	0.000
9	*TINAGL1*	Tubulointerstitial Nephritis Antigen Like 1	4.738	0.000
10	*R3HDM1*	R3H Domain Containing 1	4.711	0.013
11	*MX1*	MX Dynamin Like GTPase 1	4.468	0.000
12	*GPT2*	Glutamic–Pyruvic Transaminase 2	4.427	0.000
13	*OAS1Y*	2'-5'-Oligoadenylate Synthetase 1	4.209	0.043
14	*LRWD1*	Leucine-Rich Repeats and W.D. Repeat Domain Containing 1	4.004	0.006
15	*MX2*	MX Dynamin Like GTPase 2	3.981	0.039
16	*TRIM34*	Tripartite Motif Containing 34	3.880	0.002
17	*IRF9*	Interferon Regulatory Factor 9	3.790	0.042
18	*NDRG2*	NDRG Family Member 2	3.693	0.019
19	*SLC2A1*	Solute Carrier Family 2 Member 1	3.621	0.001
20	*TRANK1*	Tetratricopeptide Repeat and Ankyrin Repeat Containing 1	3.611	0.037

**Table 4:** Down-regulated genes in the bovine pregnant endometrium (P vs. NP)

S.N	Gene	Gene Description	Fold Change (log2)	padj
1	*SNX20*	Sorting Nexin 20	-5.182	0.014
2	*PACSIN1*	Protein Kinase C and Casein Kinase Substrate in Neurons 1	-5.086	0.022
3	*NTN3*	Netrin 3	-3.975	0.043
4	*COL1A1*	Collagen Type I Alpha 1 Chain	-2.834	0.002
5	*SLC16A4*	Solute Carrier Family 16 Member 4	-2.705	0.034
6	*TERF1*	Telomeric Repeat Binding Factor 1	-2.619	0.014
7	*NOC2L*	NOC2 Like Nucleolar Associated Transcriptional Repressor	-2.406	0.024
8	*COL3A1*	Collagen Type III Alpha 1 Chain	-2.279	0.000
9	*COL6A3*	Collagen Type VI Alpha 3 Chain	-2.219	0.042
10	*SPARCL1*	SPARC Like 1	-2.130	0.009
11	*SPARC*	Secreted Protein Acidic and Cysteine Rich	-2.128	0.001
12	*SMG6*	SMG6 Nonsense Mediated MRNA Decay Factor	-2.027	0.001

### Functional annotation and pathways enrichment analysis of DEGs (2)

The gene ontology analysis demonstrates Type-1 interferon signaling, immune response, and extracellular matrix organization were important functional pathways observed in the biological process. Similarly, ion transporters such as *SLC34A2, SLC2A1*, and *SLC16A11* were important in the molecular functions. The cellular component functions on the endoplasmic reticulum lumen were governed by genes such as *WNT5B, IL23A1*, and *PENK* (Table 5).

**Table 5:** Gene Ontology analysis in the bovine pregnant endometrium versus cyclic and nonpregnant

Gene ontology	Pregnant endometrium
Biological process	Type-1 interferon signaling (*MX1, MX2, IF16, IRF1,* and *ISG15*) Immune response (*IL23A*, and *RSAD2*) Extracellular matrix organization (*COL1A1, COL1A2, COL3A1,* and *TIMP2*)
Molecular functions	Ion transporters (*SLC34A2, SLC2A1, SLC16A11, SLC16A4* and *ATP1B1*) Platelets derived factors, telomerase activity (*HMBOX1* and *TERF1*) and ATPase activities (*P2RX6* and *DNAJB1*)
Cellular component	Endoplasmic reticulum lumen (*WNT5B, IL23A1, PENK, TNC, SPARCL1*, and *B2M*)

The pathways enrichment for the up-regulated genes in the bovine endometrium using the Kyoto Encyclopedia of Genes and Genomes (KEGG) were analyzed [24]. The official gene symbol of the up-regulated genes was uploaded to the functional annotation tool in the Enrichr system, and the bovine was selected as the reference genome. The enrichment parameters were set to a threshold gene count of 2 and a modified Fisher Exact *p*-value < 0.05. The over-represented KEGG pathways terms were considered as enriched KEGG pathways.

In the KEGG pathway, both groups (P vs. C) and (P vs. NP) had some common pathways, i.e., the mineral absorption pathway. On the other hand, Th17 cell differentiation, endocrine, and other factor-regulated calcium reabsorption and progesterone-mediated oocyte maturation pathways were highly enriched in P vs. C (Table 6), whereas extracellular matrix (ECM) receptor interactions, C-type lectin receptor signaling pathway, and IL-17 signaling pathway were enriched in P vs. NP females (Table 7). Collagen genes were found abundantly in bovine pregnant endometrium.

**Table 6:** KEGG Pathway (P vs. C)

Term	Odds Ratio	Genes
Progesterone-mediated oocyte maturation	6.23	*PDE3B, PKMYT1, ADCY6*
Th17 cell differentiation	5.50	*IL23A, FOS, JAK3*
Parathyroid hormone synthesis, secretion, and action	5.24	*SLC34A2, FOS, ADCY6*
Mineral absorption	8.49	*SLC34A2, ATP1B1*
cAMP signaling pathway	3.54	*PDE3B, FOS, ATP1B1, ADCY6*
Endocrine and other factor-regulated calcium reabsorption	6.79	*ATP1B1, ADCY6*
Regulation of lipolysis in adipocytes	6.67	*PDE3B, ADCY6*

**Table 7:** KEGG Pathway (P vs. NP)

Term	Odds Ratio	Genes
Protein digestion and absorption	9.07	*COL1A1, COL3A1, COL1A2, COL6A3*
ECM-receptor interaction	7.37	*COL1A1; COL1A2; COL6A3*
C-type lectin receptor signaling pathway	5.46	*IL23A, IRF1, IRF9*
Mineral absorption	9.27	*SLC46A1, TRPV6*
Platelet activation	4.89	*COL1A1, COL3A1, COL1A2*
IL-17 signaling pathway	4.48	*MAPK7, TRADD*
Focal adhesion	3.07	*COL1A1, COL1A2, COL6A3*
Arginine biosynthesis	10.71	*GPT2*

**Fig. 3:**
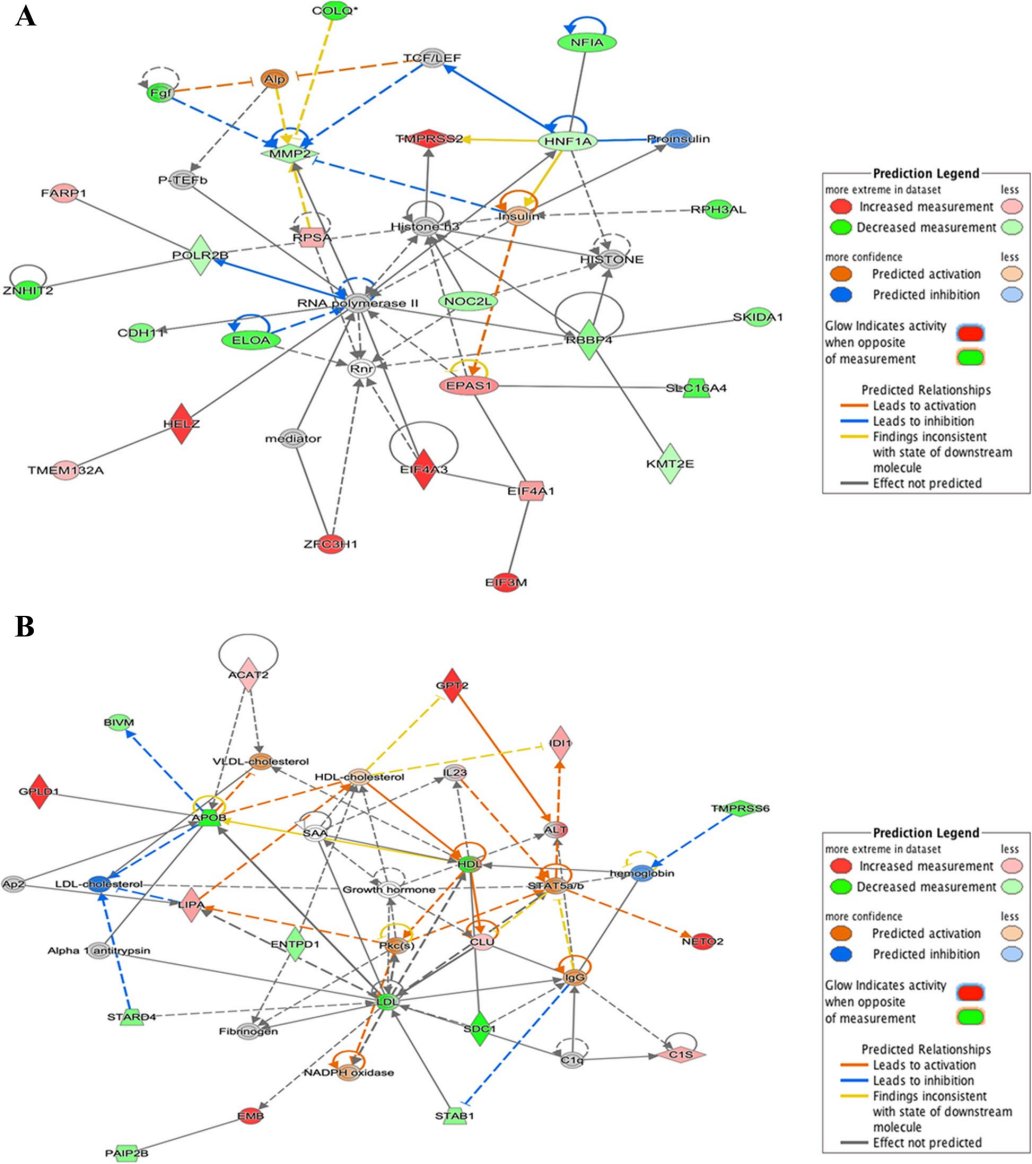
Lipid Metabolism in the bovine endometrium between **A** pregnant vs. cyclic, and **B** pregnant vs. non-pregnant. The network is displayed graphically as nodes (genes). The node color intensity indicates the expression of genes, with red representing up-regulation and green with down-regulation in the pregnant endometrium

In the ingenuity canonical pathways, two pathways were common in both groups: interferon signaling and IL-12 signaling and production in macrophages. Other uncommon pathways to the group were the Th17 activation pathway, MIF regulation of innate immunity, and IL-23 signaling pathway enriched in P vs. C (Table 8). In contrast, oxidative phosphorylation, GP6 signaling pathway, and inhibition of matrix metalloproteases were pathways that were more highly expressed in the P vs. NP females. (Table 9). In the IPA network, lipid metabolism molecules such as *Alp, CDH1, COLQ, EIF3M, EIF4A1, EIF4A3, ELOA, EPAS1, FARP1, Fgf, HELZ, HISTONE, Histoneh3, HNF1A, Insulin, KMT2E*, mediator; *MMP2, NFIA, NOC2L, PTEFb, POLR2B, Proinsulin, RBBP4, RNA polymerase II, Rnr, RPH3AL, RPSA, SKIDA1, SLC16A4, TCF/LEF, TMEM132A, TMPRSS2, ZFC3H1,* and *ZNHIT2* were significantly enriched (Fig. 3).

**Table 8:** Ingenuity Canonical Pathway (P vs. C)

Ingenuity Canonical Pathways	-log (*p*-value)	Molecules
Th17 Activation Pathway	4.40	*HIF1A, HSP90AA1, HSP90AB1, IL10, IL23A, JAK3, NFKB1, PTGER2, STAT4*
Interferon Signaling	4.38	*IFI6, IFIT1, IRF1, IRF9, ISG15, MX1*
Epithelial-Mesenchymal Transition Pathway	4.30	*EGR1, FGF14, FGF9, HIF1A, HNF1A, JAK3, MET, MMP-2, NFKB1, NOTCH4, PIK3R1, TWIST1, WNT5B*
PI3K/AKT Signaling	3.47	*CDKN1B, HSP90AA1, HSP90AB1, INPP5J, ITGA3, JAK3, NFKB1, PIK3R1, PPP2R3A, PTGS2, TP53*
IL-12 Signaling and Production in Macrophages	3.17	*APOB, FOS, IL10, IL23A, IRF1, MST1R, NFKB1, PIK3R1, STAT4*
MIF Regulation of Innate Immunity	3.03	*CD74, FOS, NFKB1, PTGS2, TP53*
CD40 Signaling	2.95	*FCER2, FOS, JAK3, NFKB1, PIK3R1, PTGS2*
IL-23 Signaling Pathway	2.94	*HIF1A, IL23A, NFKB1, PIK3R1, STAT4*
PPAR Signaling	2.55	*AIP, FOS, HSP90AA1, HSP90AB1, NFKB1, PTGS2*

**Table 9:** Ingenuity Canonical Pathway (P vs. NP)

Ingenuity Canonical Pathways	-log (*p*- value)	Molecules
Interferon Signaling	7	*IFI6, IFIT1, IRF1, IRF9, ISG15, MX1, STAT1, TAP1*
Oxidative Phosphorylation	4.09	*ATP5F1C, COX11, COX4I2, MT-CO1, MT-CYB, MT-ND1, MT-ND2, MT-ND3, MT-ND5*
GP6 Signaling Pathway	3.80	*CERT1, COL12A1, COL1A1, COL1A2, COL3A1, COL4A2, COL5A1, COL6A1, COL6A3*
Mitochondrial Dysfunction	3.25	*ATP5F1C, COX11, COX4I2, MT-CO1, MT-CYB, MT-ND1, MT-ND2, MT-ND3, MT-ND5, PDHA1*
Inhibition of Matrix Metalloproteases	2.42	*MMP-14, MMP-2, SDC1, TIMP2*
IL-12 and Macrophage production Signal	2.2	*APOB, CLU, IL23A, IRF1, STAT1, STAT4, TGFB3*

**Fig. 4:**
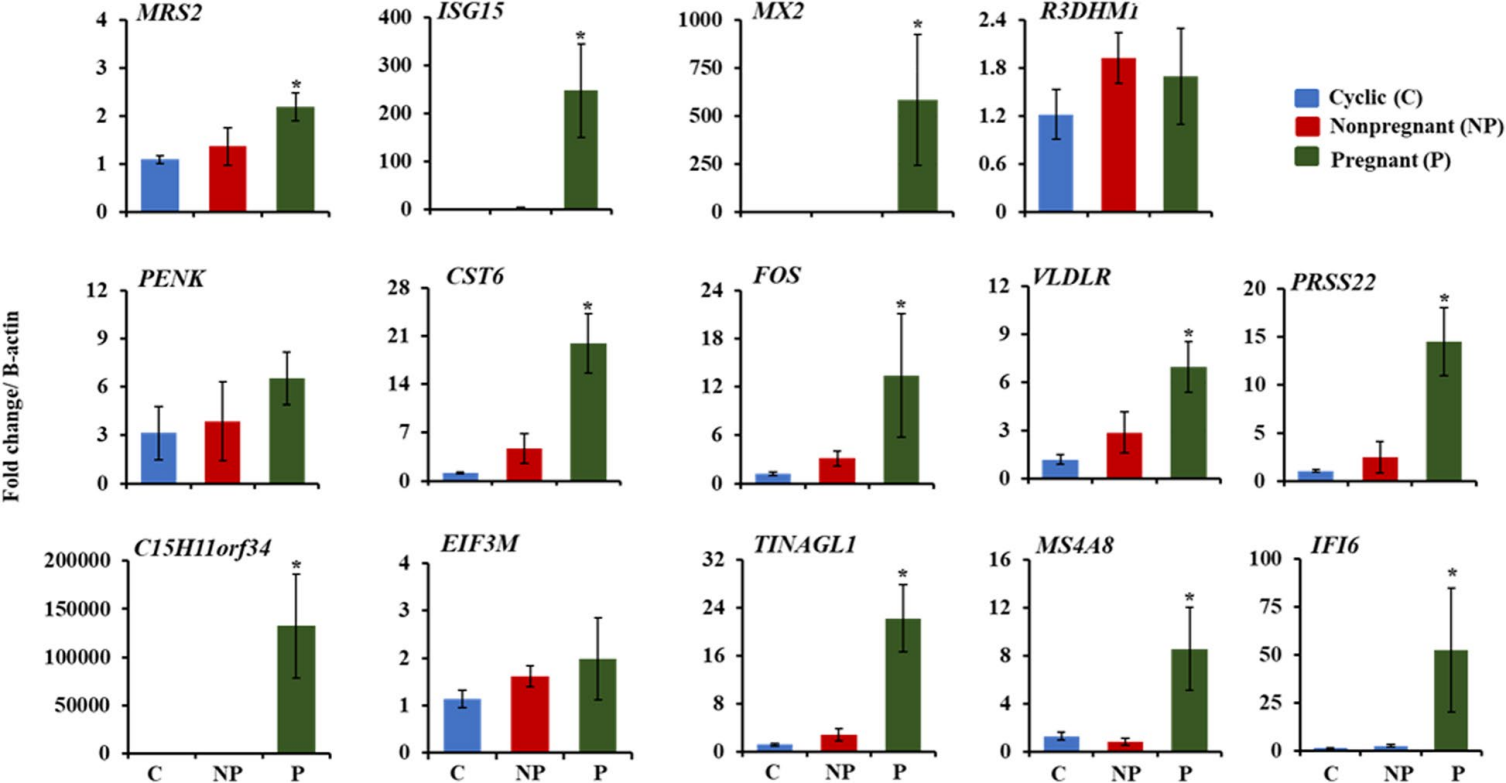
Validation of the gene expression in the endometrium. The fold changes were normalized with the *β-actin*. Data are represented as the mean ± standard error. The x-axis represents the different physiological statuses of the cows (C; cyclic, NP; non-pregnant, and P; pregnant cattle). Y-axis represents relative fold change for gene expression. All the fold change values were subjected to a one-way analysis of variance followed by the Tukey HSD test for mean separation and comparison to determine differences among the groups. Differences were considered significant at a *p-value < 0.05”

The RNA-Seq data identified the differentially expressed genes in the pregnant bovine endometrium. Among the most highly up-regulated genes, fourteen candidate genes (*MRS2, CST6, FOS, VLDLR, ISG15, IF16, MX2, C15H11ORF34, EIF3M, PENK, PRSS22, MS4A8, TINAGL1*, and *R3HDM1*) were selected for validation using qPCR. The results of relative fold change for candidate genes obtained from qPCR are shown in Fig. 4. *MRS2, MS4A8, PRSS22, CST6*, *VLDLR, IFI6, C15H11ORF34, ISG15, TINAGL1,* and *MX2* were significantly higher (*p* < 0.05) in the pregnant endometrium compared to NP and C, whereas *R3DHM1, EIF3M, FOS,* and *PENK* remained unchanged.

## Discussion

In ruminants, the successful establishment of pregnancy requires the intricate dialogue between the uterus and growing conceptus. During the maternal recognition of pregnancy, a conceptus-derived signal (IFNτ) leads to the persistence of the corpus luteum and induces the endometrial transcript to establish gestation [7]. Previous studies have identified several genes induced by IFNτ in the caruncular and intercaruncular endometrium during the peri-implantation period [16–18]. In the present study, both RNA-Seq and qPCR analysis confirmed the differential expression of several pre-discovered and novel genes and their biological pathways in the pregnant endometrium compared to cyclic and non-pregnant endometrium. Interferon signaling, immune response, nutrient transporter, synthesis, and secretion of proteins are crucial pathways during the maternal recognition of pregnancy. This study found some important molecules such as Type-1 interferon signaling *(MX1, MX2, IF16, IRF1*, and *ISG15)*, ion transporters (*SLC34A2, SLC2A1, SLC16A11,* and *SLC16A4*), ECM organization (*COL1A1, COL1A2,* and *COL3A1*), and novel genes (*MRS2, C15H11ORF34*).

### Interferon signaling pathway

The interferon signaling pathway is important in the pregnant endometrium around the peri-implantation period [7, 21]. In this study, the interferon signaling pathway was highly enriched in the pregnant endometrium. Under interferon signaling, we identified several genes such as *MX2, IFI6, IFIT1, IRF1, IRF9, ISG15, MX1, STAT1*, and *TAP1*. *ISG15* is among the highly up-regulated genes in the pregnant endometrium. Gene Ontology and Ingenuity pathway analysis from our study revealed that *ISG15* functions in the interferon signaling activation pathway in the pregnant endometrium, which is very important for the maternal recognition of the pregnancy. Previous studies have detected the *ISG15* expression in the stromal cells and glandular epithelial cells [6, 12]. *ISG15* expression is linked with several activities such as gene transcription, DNA repair, signal transduction, apoptosis, and cell cycle by conjugating itself to those target proteins [26]. *ISG15* can regulate the innate immunity of embryonic cells. *IFI6* was highly up-regulated in the pregnant endometrium and is part of the interferon signaling pathway. *IFI6* is not mapped yet in cattle, but the human *IFI6* gene is located on HSAP1p35, homologous to a chromosomal region between cattle and humans [27]. The timing of the up-regulation of ISGs, such as *IFI6* in pregnant heifers was observed in previous studies [12]. Our study and previously reported suggest that *IFI6* signaling is required to maintain the endometrial immune status required for embryonic survival and maternal recognition of pregnancy. In the present study, *MX2* was among the highly up-regulated genes. *MX2*, which is recognized as an intracellular antiviral protein, belongs to a large GTPase family. It takes part in the interferon signaling pathway and innate immune system. In our study, GO annotations related to this gene include GTP binding and GTPase activity. *MX2* is up-regulated in response to IFNτ from the elongating conceptus [7]. *MX2* expression in response to elongating conceptus is consistent across mammalian species, including cattle, sheep, and humans. The *MX2* expression was increased in the pregnant endometrium of ewes [28] and cows [29] in response to IFNτ. It is reported that *MX2* mRNA is detectable in the peripheral blood lymphocytes with higher intensity at Day 16 of gestation than in non-pregnant cows [29]. Further, gene ontology analysis suggested that *MX2* is an important antiviral gene in the uterus during the preimplantation period.

### Extracellular matrix signaling

Extensive extracellular matrix and cellular remodeling occur in the endometrium during the estrous cycle, peri-implantation period, and different gestation stages [20, 30–34]. Collagen, the most abundant extracellular protein in mammals, is the main structural protein in the extracellular matrix in the various connective tissues [33]. In this study, GO analysis detected several collagen genes (*COL1A1, COL1A2,* and *COL3A1*) involved in the extracellular matrix organization in the pregnant bovine endometrium. We also found that endometrial collagen genes are predicted in platelet-derived growth factor(s). The KEGG pathway revealed many collagen genes in the pregnant endometrium having different functions. The genes involved in protein digestion and absorption (*COL1A1, COL3A1, COL1A2*, and *COL6A3)*, ECM-receptor interaction *(COL1A1, COL1A2*, and *COL6A3),* and platelet activation (*COL1A1, COL3A1,* and *COL1A2)*. Collagen genes have an essential role in cell adhesion. Some of the important collagen genes that help in adhesion are *COL1A1, COL1A2,* and *COL6A3*. According to our findings from the Ingenuity Canonical Pathways analysis, *COL12A1, COL1A1, COL1A2, COL3A1, COL4A2, COL5A1, COL6A1*, and *COL6A3* are involved in the Glycoprotein 6 (GP 6) signaling pathway.

Matrix metalloproteinases (MMP) are known to degrade the ECM for cellular proliferation, differentiation, migration, and apoptosis [30–34]. In our study, ingenuity canonical pathway identified the genes (*MMP-2, MMP-14, SDC1, TIMP2)*. It is well-known that MMP-14 regulates the *MMP-2* through binding *TIMP-2*. This MMP cascade regulated the endometrial cell functions required for embryo implantation [30–34]. Syndecan-1 (SDC1/CD138)) is an integral membrane protein and takes part in cell proliferation, cell migration, and cell–matrix interactions [35]. Although cyclic and non-pregnant cows lacked a conceptus, interestingly, in our study, we found some molecules such as COL1A1, TRADD, and COL3A1 were up-regulated in both non-pregnant and pregnant endometrium. One reason could be that those collagen genes (*COL1A1* and *COL3A1*) are abundantly present in platelet-derived growth factor binding [36]. In contrast, TRADD is essential for death-inducing signaling, which is likely a pathway important for pregnancy and nonpregnancy in cattle [37].

Cystatin 6 or Cystatin E/M (*CST6*) is among the highly up-regulated genes in the pregnant endometrium. GO annotations related to this gene include cysteine-type endopeptidase inhibitor activity. The functions of the *CST6* include uterine endometrial and placental tissue remodeling and facilitating transplacental transport of nutrients. *CST6* expression was detected in the endometrium during the estrous cycle and pregnancy. The expression of *CST6* in chorionic epithelia of the placental membrane was increasing during late pregnancy, suggesting that cell type-specific expression and function of *CST6* are critical for appropriate maternal–fetal interaction [38].

### Ions transporter signaling pathways

Solute carrier (SLC) genes, consisting of 52 families, are mostly located in the cell membrane and code for membrane transport proteins [39]. The function of the SLC gene includes the transport of glucose, electrolytes, and amino acids. Since numerous nutrients and electrolytes must be transported from the blood to the uterine environment, SLC genes play a critical role in the bovine endometrium. *SLC2A1* was among the top 20 most up-regulated SLC genes in the pregnant endometrium. Conversely, *SLC16A11* (monocarboxylate transporter), *SLC16A4* (monocarboxylate transporter), and *SLC7A4* (cationic amino acid transporter/glycoprotein- associated amino acid) were found to be down-regulated in the pregnant endometrium. *SLC46A1* was up-regulated in the non-pregnant endometrium. GO analysis showed *SLC34A2* (Type-II Na + /HPO42- co-transporter), *SLC2A1*(glucose transporter), *SLC16A11* (monocarboxylate transporter), and *SLC16A4* (monocarboxylate transporter) involved in ion transportation [40]. *SLC34A2* was included in parathyroid hormone synthesis, secretion, and action. This gene was also found in the mineral absorption pathway. The *SLC46A1* (folic acid transporter) was also in the pregnant bovine endometrium. These results suggest that transporter molecules transport nutrients from blood circulation to the endometrial cells for their growth and development and then transported to the uterine lumen to nourish the embryo. Magnesium Transporter *MRS2* (*MRS2*) is one of the highly up-regulated genes in the pregnant endometrium. *MRS2* is a protein-coding gene located in mitochondria [41]. The pathway analysis from our result showed that *MRS2* plays a significant role in the cell cycle, cellular assembly, and organization while having a significant role in DNA replication, recombination, and repair. According to GO analysis, MRS2 is associated with magnesium ion transportation. For the first time, this study reported *MRS2* in the bovine endometrium during the maternal recognition of pregnancy. However, the spatiotemporal expression of *MRS2* is completely unknown in the bovine endometrium.

### Immunity and inflammation signaling pathways

Immunity and inflammation play a vital role in the endometrium during implantation, placentation, and fetal development [42]. The developing embryo modulates the maternal immune system and promotes maternal tolerance to the embryo during the peri-implantation period [43]. T helper 17 (Th17) cells produce IL-17 which recruits neutrophils via granulocyte colony-stimulating factor and IL-8 [44]. Th17 cells are known to regulate the inflammatory process in the endometrium required for the establishment and maintenance of pregnancy [45]. Non-pregnant women with unexplained recurrent pregnancy loss had a higher proportion of Th17 cell levels in the circulating blood than parous controls [45]. *FOS* is among the highly up-regulated gene in pregnant bovine endometrium. Ingenuity canonical pathway analysis from this study revealed that *FOS* impacts IL-12 signaling, and macrophage production has an important immune function and regulates innate immunity. It acts on the cAMP signaling pathway. Furthermore, the KEGG pathway shows that *FOS* takes part in endocrine activities such as parathyroid hormone synthesis, secretion, and action, and it has a significant role in Th17 cell differentiation. GO annotations related to this gene include DNA-binding transcription factor activity. The altered expression and distribution of *FOS* protein prompted endometriosis in the baboon [46].

### Early-pregnancy associated novel genes in the bovine endometrium

Very Low-Density Lipoprotein Receptor (*VLDLR*) is highly up-regulated in the pregnant endometrium. *VLDLR* belongs to the low-density-lipoprotein (LDL) transmembrane receptor family that localizes to the plasma membrane and is located at chromosome 9 [47]. *VLDLR* consists of cell surface proteins involved in receptor-mediated endocytosis of specific ligands. This gene encodes a lipoprotein receptor, a member of the LDLR family, and plays a vital role in VLDL-triglyceride metabolism. *VLDLR* is considered as a potential mediator of P4-dependent signaling through membrane progesterone receptors (mPR) to drive oocyte maturation and meiosis progression. It was found that the knocking down of *VLDLR* inhibited the oocyte maturation and meiosis progression. In contrast, overexpression of the *VLDLR* showed the exact opposite action of what it did when it was knocked down, confirming its importance on P4-dependent oocyte maturation [48]. *VLDLR* is known to permit cholesterol to reach tissues from the bloodstream, and it may be used as an energy source. Endometrial cells secrete large amounts of cytokines, growth factors, and other molecules for embryonic growth and development during pregnancy. Therefore, *VLDLR* might play an important role in endometrial cell function for the establishment of pregnancy.

*C15H11ORF34*, also known as Placenta Expressed Transcript 1 (PLET1), is highly up-regulated in the pregnant endometrium. RNA-Seq data identified unannotated genes such as *C15H11ORF34* up-regulated in embryos derived from T-cells [49]. This gene was among the top 10 up-regulated genes in our study, and the qPCR validation result showed that it was the most highly expressed gene in the pregnant bovine endometrium. The spatiotemporal expression and function of this gene are entirely unknown. Eukaryotic Translation Initiation Factor 3 Subunit M (*EIF3M*) is among the up-regulated genes in the pregnant endometrium. *EIF3M* is found in the cytosol [23, 50]. *EIF3M* functions in lipid metabolism, molecular transport, and protein synthesis. Furthermore, it is also involved in cell cycling, cell morphology, and apoptosis. IPA analysis from the study shows it is associated with the regulation of eIF4 (Eukaryotic Initiation Factor-4) signaling.

*TINAGL1* is among the highly up-regulated gene in the pregnant endometrium. *TINAGL1* is important in antimicrobial response, cell signaling, and inflammatory response. A previous study has shown an increased expression of *TINAGL1* on Day-13 in pregnant heifers [26]

Proenkephalin (*PENK*) is a highly up-regulated gene in the pregnant endometrium. *PENK* is a member of the opioid polypeptide hormone found in various mammals, rodents, and avian species [51, 52]. It is known to play a role in many physiologic functions, including pain perception and stress responses. The previous study showed an increased *PENK* expression on Day 13 in heifers’ endometrium [26]. Besides its dominance in the CNS, it is also expressed in the oviducts in chicken and is associated with eggshell calcification [51, 52]. However, its mechanistic role in the bovine uterus has not been established. *PENK* was detected in the myometrial region of the pregnant mouse's uterus until Day 18 of pregnancy and helped in maternal adaptation to pregnancy and supporting embryo growth. *PENK* detected in the uterus was suggested to have multiple material adaptation roles to pregnancy and support embryo growth [53].

*PRSS22* is a highly up-regulated gene in the pregnant endometrium. *PRSS22* is located on chromosome 16 and has a predicted function related to serine-type endopeptidase activity. *PRSS22* has been reported in the endometrial region of mice and humans [54] but has not previously been reported in cattle. *MS4A8* is highly up-regulated in the pregnant bovine endometrium. It has membranous and cytoplasmic gene expression, especially in Fallopian tubes and respiratory epithelium in humans [55]. *R3HDM1* is among the up-regulated gene in the pregnant endometrium. According to Entrez Gene, R*3HDM1* maps on chromosome 2 at 2q21.3. GO annotations related to this gene include nucleic acid-binding.

In conclusion, our study showed a significant difference in gene expression between pregnant versus cyclic or non-pregnant cows. The presence of an embryo induces the endometrial transcripts related to endometrial remodeling, immune response, nutrient, ion transporters, and relevant signaling pathways in the caruncular region of bovine endometrial tissue. Further, in the absence of the embryo, these transcripts are downregulated in the endometrium. In this study, using RNA-sequencing, we found some novel genes (*MRS2*, *C15H11ORF34*, *VLDLR*, and *PRSS22*). This study provides a comprehensive dataset of transcript changes associated with maternal recognition of pregnancy, which can further be linked with the specific functions of the identified genes for future experimentation.

## Supplementary Information

**Additional file 1: Supplementary Table S1.** Primers used to quantify the expression of target genes by qPCR.

## Data Availability

The datasets used and/or analyzed during the current study are available from the corresponding author on reasonable request. RNA-Sequencing data were submitted to the NCBI GEO repository (GSE196789) https://www.ncbi.nlm.nih.gov/geo/query/acc.cgi?acc=GSE196789.
