# Supplementary material for: RNA-Sequencing based analysis of bovine endometrium during the maternal recognition of pregnancy
Source: BMC Genomics. 2022 Jul 7;23:494. doi: 10.1186/s12864-022-08720-4 (PMC9264496; doi:10.1186/s12864-022-08720-4)
Supplement: Supplementary file 1 — Additional file 1: Supplementary Table S1. Primers used to quantify the expression of target genes by qPCR. [file 12864_2022_8720_MOESM1_ESM.docx]

**Supplementary Table S1**: Primers used to quantify the expression of target genes by qPCR.

| S.N. | Gene | Accession no. | Primer Sequence | Amplicon (bp) |
| --- | --- | --- | --- | --- |
| 1 | *MRS2* | NM_001101903.1 | F: GGGATTGACCATGCAGAGGA  R: CACATTACGGTGGCTGTCCA | 150 |
| 2 | *CST6* | NM_001012764.3 | F: CAAGTACTACCTGACCGTGGAC  R: CACAGCGCAGCTTCTCCT | 125 |
| 3 | *FOS* | NM_182786.2 | F: AAAGGCGAATCCGAAGGGAA  R: AGTTGGTCTGTCTCCGCTTG | 100 |
| 4 | *VLDLR* | NM_174489.2 | F: TGTGCAAGGCAGTAGGCAAA  R: CAGCGATGTCAGCATCGAGA | 141 |
| 5 | *IFI6* | XM_002685877.4 | F:GTCAAGGATACACCTGTGAAGAAAA  R: GGAGTCTGAAGAAGGCCCTTAG | 140 |
| 6 | *MX2* | NM_173941.2 | F: GCCCGCCATTGCCGTTA  R: CCGGGTGATGATTCCGCTG | 103 |
| 7 | *C15H11ORF34* | NM_001113538.1 | F: AGCACGCTCTTCAAGGCAAA  R: GACGGTCACAGTCCCAACTT | 141 |
| 8 | *EIFM3* | XM_010812722.2 | F: TGGGAATGGCCGTGGAAAAT  R: TCCGATGTGTGCTATGACATCAA | 113 |
| 9 | *TINAGL1* | XM_015459968.2 | F: CACGGCAGCTGTGGCA  R: CACCAGGCACCATCCAGTC | 141 |
| 10 | *ISG15* | NM_174366.1 | F: GCAGACCAGTTCTGGCTGTCT  R: CCAGCGGGTGCTCATCAT | 140 |
| 11 | *PENK* | NM_174141.2 | F: GAACAGCGGCAACCCCAT  R: GCAGTCCTGGCTGCATTCT | 149 |
| 12 | *PRSS22* | XM_002697927.5 | F: CTATCAAGACAGCCGGCCC  R: TCTTCCGGATGCTCACAACC | 114 |
| 13 | *MS4A8* | NM_001034056.2 | F: GGGGAGGCATCTGGTTCATC  R: GACGATGTTAAAGCCCACGC | 144 |
| 14 | *R3DHM1* | XM_024980920.1 | F: GTCATTCCACCTGGCCAACA  R: GTGGAGGTGGCGCTGC | 148 |
| 15 | *β-ACTIN* | NM_173979.3 | F: GAAGATCAAGATCATCGCGCC  R: GTGTAACGCAGCTAACAGTC | 177 |
